# The impact of the COVID-19 pandemic on time to treatment in head and neck cancer management: a systematic review

**DOI:** 10.2340/1651-226X.2025.41366

**Published:** 2025-01-28

**Authors:** Malte Grumstrup Simonsen, Amanda-Louise Fenger Carlander, Kathrine Kronberg Jakobsen, Christian Grønhøj, Christian von Buchwald

**Affiliations:** a Department of Otolaryngology, Head and Neck Surgery and Audiology, Rigshospitalet, Copenhagen University Hospital, Copenhagen, Denmark; b Department of Clinical Medicine, University of Copenhagen, Copenhagen, Denmark

**Keywords:** Time to treatment initiation, tumor stage, delay, waiting time

## Abstract

**Background and purpose:**

Coronavirus disease 2019 (COVID-19) caused a need for reorganization in the healthcare systems. First, we aimed to determine the impact of the COVID-19 pandemic on time to treatment in head and neck cancer (HNC) patients. Second, we aimed to determine the impact of COVID-19 on tumor stage and changes in treatment regimens used.

**Material and methods:**

A systematic search in PubMed and Embase was conducted according to the Preferred Reporting Items for Systematic Reviews and Meta-Analyses guidelines. Inclusion criteria were: (1) Studies including patients with head and neck squamous cell carcinomas; (2) Studies containing a comparison of time to treatment; (3) Studies containing a well-defined time interval with restrictions on health care due to COVID-19 and a well-defined time interval without restrictions.

**Results:**

A total of 19 studies were included comprising 24,898 patients treated for HNC cancer. Six studies (10.1% of the patients) reported an increase in waiting time within at least one interval, while seven studies reported a decrease (83.2% of the patients), and six studies found no significant effect. No changes in treatment modalities were observed. Seven of 15 studies (12.7% of the patients) observed an increase in either overall stage, size, or tumor node and metastasis classification during the COVID-19 pandemic. Among these, two studies reported increased waiting times as well.

**Interpretation:**

The impact of the COIVD-19 pandemic on time to treatment was heterogenous and subject to considerable intercountry and interregional variations. A tendency toward a higher T-classification was observed. In conclusion, otorhinolaryngology departments demonstrated resilience, as the pandemic led to only slight alterations in time to treatment.

## Introduction

Coronavirus disease 2019 (COVID-19) caused a profound need for reorganization in the healthcare systems worldwide. The prompt global spread led to the World Health Organization (WHO) declaring the virus a pandemic on the 11^th^ of March 2020 [1]. Globally, resources were reallocated toward the prevention and care of COVID-19 patients, potentially impacting the availability of diagnostics and treatment of other diseases [2–5].

The management of head and neck cancer (HNC) patients underwent comprehensive evaluation, given the transmission of COVID-19 primarily through the nasal and respiratory pathways [6]. Guidelines regarding medical care of HNC patients were made, including recommendations for the management of potential treatment delays [7, 8]. Along with the reduction in elective procedures on medical care centers [9, 10], many dental clinics closed during the early stages of the pandemic, removing an important healthcare provider [11]. Diversion of resources and the increased risk of exposure to COVID-19 for patients seeking medical care raised concerns of increases in time to treatment in HNC [8, 12].

Studies indicate that increases in time to the treatment of HNC patients are associated with a higher tumor stage and worse survival, although the results have been inconsistent, possibly due to large heterogeneities in study designs and definitions of treatment delay [13, 14].

The aim of this systematic review was to determine the impact of the COVID-19 pandemic on time to treatment in HNC patients as well as to elucidate the impact of COVID-19 on tumor stage and treatment regimens used.

## Methods and materials

This systematic review followed the 2020 Preferred Reporting Items for Systematic Reviews and Meta-Analyses (PRISMA) guidelines [15].

### Search strategy

A systematic search was conducted in PubMed and Embase with the final search being on 13th of October 2023. Two authors (MG and ALFC) independently screened the studies eligible for inclusion.

The following keywords were identified: ‘Time to treatment’ and ‘head and neck squamous cell carcinomas’, and they were subsequently assigned to their corresponding MeSH-term (PubMed) or emtree-term (Embase). For completeness, synonyms of the keywords were also included in the final search. With the exposure of the study being the COVID-19 pandemic, publication year was set to be not earlier than January 2020. The full search can be found in the supplementary material.

### Eligibility criteria

Full-text studies were included according to the following criteria: (1) Studies including patients with head and neck squamous cell carcinomas (HNSCC), (2) Studies containing a comparison of time to treatment, and (3) Studies containing a well-defined time interval with restrictions on health care due to COVID-19 and a well-defined time interval without.

Studies were excluded if there was no measurement of time to treatment, no comparison between a COVID-19 and a non-COVID-19 group, less than 10 participants, no data specifically on HNC, and no full-text was available. Studies not published in Danish, Norwegian, Swedish, or English were also excluded.

### Data items

The subsequent data were retrieved: Author, publication year, geographical location of study population, study period, age, number of patients, definition of time to treatment, tumor sites, treatment modality used, oncological outcome (Tumor Node and Metastasis [TNM] classification, changes in Union for International Cancer Control (UICC) stage grouping or changes in mean tumor size), and time to treatment including a definition of the time interval measured.

In this review, the term ‘time to treatment’ was used to describe any interval from the debut of symptoms until the beginning of therapy. ‘Symptom’ was defined as the first day of symptoms, as reported by the patient. ‘Specialist’ was defined as the first visit to the respective healthcare center, which determines diagnosis and initiates treatment.

### Assessment of outcomes

Reporting quality and risk of bias was assessed using the 20 component AXIS-tool for cross sectional studies [16]. Appraisal was done by one researcher (MGS) (Supplementary material for details).

## Results

### Study selection

**Figure 1:**
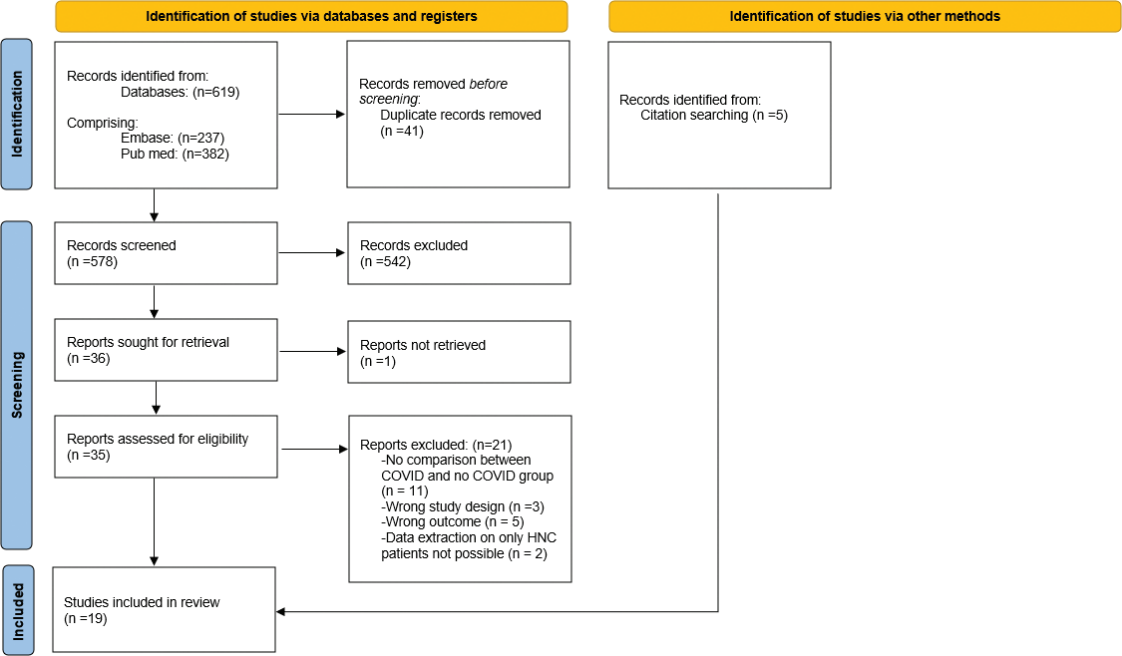
Preferred Reporting Items for Systematic Reviews and Meta-Analyses flow of study selection.

The literature search yielded 578 results after removal of duplicates. A total of 36 full texts were assessed for eligibility, with 14 studies meeting the inclusion criteria [17–30]. Additionally, five studies were identified through screening of references [31–35]. A total of 19 studies were enrolled [17–35] (see Figure 1).

### Study characteristics

A total of 24,898 patients were included. Median number of patients in the study was 265 (range: 49–10,880). The types of HNCs assessed were: Ten studies reported on all the HNCs [18, 19, 20, 23, 25, 27–30, 33], three studies looked at specifically HNSCC [24, 32, 35], while six studies only assessed either sinonasal, nasopharyngeal, oral, or laryngeal cancer [17, 21, 22, 26, 31, 34]. Twelve studies analyzed data from a single tertiary center [17–19, 21, 22, 24, 26, 30, 31, 32, 33, 35], while seven studies obtained data from a register [20, 23, 25, 27–29, 34]. Geographic locations included: Croatia [17], Germany [25, 26, 34, 35], England [18], Italy [19], the Netherlands [20], Scotland [29], Switzerland [21], Turkey [22], Wales [27], Canada [23], the United States [24, 28, 30, 32, 33], and China [31]. Median age of the patients was 64.5 years (range: 50.5–72.5 years). Median male to female ratio was 2.4 (range: 1.2–10.2). Periods defined as ‘non-COVID-19’ and ‘COVID-19’ varied between studies, with some [19, 20, 22–24, 27–35] choosing an interval within a lockdown period from the respective country as a marker of the COVID-19 period, and others [17, 18, 21, 25, 26, 28] defining January 2020 as the beginning of the COVID-19 period. Treatment was either surgery, radiotherapy, chemotherapy, chemoradiotherapy, or a combination. A full overview of study characteristics is shown in Table 1.

**Table 1:** Overview of study characteristics.

Authors, country of the study, publication year	Centre/database	Study period (non-COVID-19)	Study period (COVID-19)	Time to treatment intervals	No. of patients	Age*	M/F ratio(s)**	Site	Outcomes
***Europe***									
Gršić, Croatia, 2022	Zagreb University Hospital	2018 + 2019	2020 + 2021	Symptoms to specialist	691	61.1; 66.4	1.5; 10.2	Oral, Larynx	Time to treatment Clinical TNM classification UICC overall stage
Zubair, England, 2022	Royal London Hospital	January to October, 2019	January to October, 2020	Referral to specialist Referral to treatment Diagnosis to treatment	104	N.A.	N.A.	HNC	Time to treatment UICC overall stage
Heckel, Germany, 2023	UCC-R (Eastern part of Bavaria)	2019	2020	Diagnosis to treatment	706	63.0	2.3; 2.8	HNC	Time to treatment Clinical TNM classification Pathological TNM classification UICC overall stage
Metzger, Germany, 2021	Heidelberg University Hospital	2010–2019	2020	Specialist to treatment	624	65	1.4	Oral cancer	Time to treatment Pathological TNM classification UICC overall stage
Kourtidis, Germany, 2022	Charité Hospital, Berlin ENT surgery dept.	March 2019 to March 2020	March 2020 to March 2021***	Symptom to diagnosis Diagnosis to treatment	94	67.4; 69	2.4; 3.3	HNSCC	Time to treatment Clinical TNM classification
Heimes, Germany, 2021	Maxillofacial departments of Kiel, Mainz, and Berlin	June to November, 2018 June to November, 2019	March to June, 2020***	Time to intervention	653	N.A.	N.A.	Oral cancer	Time to treatment T and N classification UICC overall stage
Lucidi, Italy, 2022	University Hospital of Modena, Italy	March to October, 2019	March to October, 2020***	Specialist to treatment	265	66.4; 68.5	N.A.	HNC	Time to treatment UICC overall stage
Schoonbeek, Netherlands, 2021	Netherlands Cancer Registry	March to June, 2018 March to June, 2019	March to June, 2020***	Specialist to treatment Biopsy to treatment	8468	66.1 ; 66.4	1.7 ; 1.9	HNC	Time to treatment UICC overall stage
Drake, Scotland, 2022	MDT data from West of Scotland	March to May, 2019	March to May, 2020***	Referral to diagnosis Referral to treatment	236	61.5 ; 63.7	2.1 ; 3.4	HNC	Time to treatment
Meerwein, Switzerland, 2021	University Hospital, Zürich	2018 + 2019	2020	Symptom to biopsy Symptom to treatment Referral to treatment	49	66	1.6	Sinonasal Nasopharynx	Time to treatment Clinical TNM classification UICC overall stage
Tevetoglu, Turkey, 2021	Cerraphasa Medical Faculty, Istanbul	March to September, 2019	March to September, 2020***	Symptom to specialist Specialist to treatment	116	60.3 ; 64.3	4.5 ; 6	Oral, Larynx	Time to treatment T and N classification
Abelardo, Wales, 2022	Hywe Dda University Health Board	April to November, 2020	April to November, 2020***	Referral to specialist Referral to MDT Referral to treatment	143	72 ; 72.5	2	HNC	Time to treatment
***Northern America***									
Psycharis, Canada, 2023	Cancer and diagnosis committee’s database of the McGill University Health Centre Cancer Registry	July 2019 to February 2020	March to October, 2020***	Specialist to MDT Specialist to treatment Biopsy to diagnosis	265	57 ; 61	2.2 ; 2.8	HNC	Time to treatment TNM classification
Solis, USA, 2021	University of California, Davis, ENT Surgery Department	September 2019 to March 2020	March to September, 2020***	Symptom to specialist Biopsy to surgery Specialist to surgery Scan to treatment Diagnosis to first visit	137	65.5	2.5	HNSCC	Time to treatment TNM classification Median tumor size
Yao, USA, 2021	Tertiary Academic Medical Hospital in New York City	September 2019 to January 2020	March to July, 2020***	Suspicion to diagnosis Suspicion to stageing Diagnosis to treatment	94	64	1.2	HNC	Time to treatment
Kiong, USA, 2022	University of Texas M.D. Anderson Cancer Center	May to June, 2019	May to June, 2019	Symptom to specialist Diagnosis to first visit First visit to MDT	231	65	3.2	HNC	Time to treatment TNM classification Median tumor size UICC overall stage
Tasoulas, USA, 2023	National cancer database (NCDB)	2019	2020	Diagnosis to treatment	10880 (in 2020)	64 (in 2020)	1.9 (in 2020)	HNC	Time to treatment
Stevens, USA, 2022	Vanderbilt University Medical Center	March to July, 2019	March to July, 2020***	Referral to specialist Symptom to specialist	268	62.9 ; 64.5	3.1 ; 2.7	HNSCC	Time to treatment Clinical TNM classification Pathological TNM classification Upstaging (c < p)
***Asia***									
Yang, China, 2020	Fudan University Shanghai Cancer Center	December 2019 to January 2020	January to February, 2020***	Pathological consultation report Report from biopsies Imaging examination Radiotherapy immobilization and simulation Validation of position and plan Initiation of treatment	874	50.5	2.7 ; 3	Nasopharynx	Time to treatment UICC overall stage

This table shows the baseline characteristics of the studies included.

N.A.: data not available; HNC: all head and neck cancers; HNSCC: head and neck squamous cell carcinomas; No: number; M/F: male/female; MDT: multidisciplinary team conference; TNM: Tumor, Node and Metastasis; UICC: Union for International Cancer Control; COVID-19: coronavirus disease 2019.

* Data on age are separated with a ‘;’ when more than one average age is presented. The first value indicated corresponds to the non-COVID-19 group, and the second value to the COVID-19 group.

** M/F ratios are separated with a ‘;’ when more than one M/F ratio is presented. The first value indicated corresponds to the non-COVID-19 group, and the second value to the COVID-19 group.

*** COVID-19 period is within a lockdown period from the respective country.

### Time to treatment intervals

**Figure 2:**
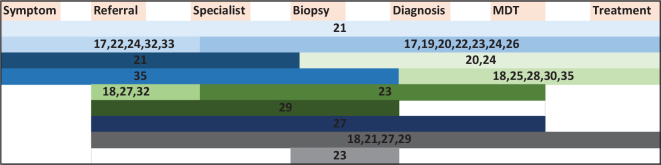
Intervals investigated in included studies from the onset of symptoms to initiation of treatment. Each study is referenced with their corresponding reference number. The length of each bar represents a specific interval, and each bar corresponds to only one interval.

A total of 13 different time intervals were reported, encompassing the period from onset of symptoms to initiation of treatment, see Figure 2. Five studies [24, 30, 31, 33, 34] used intervals that did not fit in the intervals mentioned in the figure. Heimes et al. analyzed ‘time to intervention’ [34], Yao et al. reported on intervals starting from initial documented suspicion of cancer [30], Yang et al. analyzed time to treatment in each step in a pathway from diagnosis to treatment [31], and Kiong [33] and Solis [24] included the interval between the patient’s initial diagnosis at another medical center and their first appointment at Kiong and Solis’ respective centers.

### Time to treatment

Six studies found no significant difference in time to treatment across all intervals investigated (*n* = 1,616) [19, 24, 29, 33–35].

Six studies found a significant increase in time to treatment in the COVID-19 group within at least one interval (*n* = 2,503) [17, 18, 22, 26, 30, 31]. Increases in days from specialist to initiation of treatment were observed in two studies [17, 26]. Gršić et al. observed an average increase of 11 days (26 days vs 37 days, *p* = 0.006) and 10 days (21.5 days vs 31.5 days, *p* = 0.001) for patients with oral and laryngeal cancer, respectively (*n* = 691) [17]. Similarly, Metzger et al. identified an average increase of 10 days (35 days vs 45 days, *p* = 0.04) across all HNCs (*n* = 624) [26]. Additionally, both Gršić et al. and Tevetoğlu et al. (*n* = 116) found an increase in the symptom to specialist interval for oral cancer of 22.5 days (37.5 days vs 60 days, *p* = 0.019) [17] and 2.4 days (16.6 days vs 19.0 days, *p* = 0.02), respectively [22].

Zubair et al. investigated the interval from referral to initiation of treatment and found an increase of 23.3 days in the COVID-19 group compared to the non-COVID-19 group (49.2 days vs 72.5 days, *p* = 0.027) (*n* = 104) [18]. Yao et al. reported, among other intervals, on the time from first documentation of cancer suspicion to diagnosis and observed that patients in the COVID-19 group had a significantly longer time to diagnosis than the non-COVID-19 group (hazard ratio: 0.54, *p* = 0.02) (*n* = 94) [30]. Yang et al. identified significant increases in days in the COVID-19 group regarding waiting time for: pathological biopsy (5 days vs 15 days, *p* = 0.012), radiotherapy immobilization and simulation (3.5 days vs 16.5 days, *p* < 0.001), validation of position and plan (20 days vs 61 days, *p* < 0.001), and initiation of radiotherapy (28 days vs 36 days, *p* = 0.005) (*n* = 874) [31]. The median duration of increased time to treatment across studies was 11 days, with intervals ranging from 7 to 41 days. In total, increased time to treatment was observed in Croatia [17], Germany [26], England [18], Turkey [22], the United States [30], and China [31].

Seven studies found a significant decrease in time to treatment in the COVID-19 group within at least one interval (*n* = 20,779) [20, 21, 23, 25, 27, 28, 32]. A decrease of 5 days from specialist to treatment was observed by Schoonbeek et al. (31 days vs 26 days, *p* < 0.001) (*n* = 8468) [20]. In addition, a decrease in time from the date of biopsy to treatment was also found (37 days vs 30 days, *p* < 0.01) [20]. Psychiaris et al. found a decrease of 27.9 days from specialist to treatment (76.6 days vs 48.7 days, *p* > 0.01) (*n* = 265) [23]. They also found a decrease of 12.9 days in the interval from specialist to presentation at multidisciplinary team (MDT) conference in the COVID-19 group compared to the non-COVID-19 group (38 days vs 25.1 days, *p* = 0.0001) [23].

Two studies found a decrease in the interval from diagnosis to initiation of treatment [25, 28]. Heckel et al. found a decrease of 3.5 days in the COVID-19 group (23 days vs 19.5 days, *p* = 0.013) [25], while Tasoulas et al. found a decrease of 3 days decrease (46 days [95% CI: 46–47] days vs 43 [95% CI: 42–43]) (*n* = 10,880) [28].

Two studies found a decrease in the period from referral to specialist. Abelardo et al. found a decrease of one and a half days in the COVID-19 group (9.5 days vs 8 days, *p* > 0.01) (*n* = 143) [27]. Stevens et al. found a decrease of 3 days (11 days vs 8 days, *p* = 0.008) [32], and Meerwein et al. found a 7-day decrease from referral to initiation of treatment (18 days vs 11 days, *p* = 0.02, *n* = 49) [21]. The median duration of decreased time to treatment across studies was 5 days, with intervals ranging from 1.5 to 28 days. In total, decreased time to treatment was found in the Netherlands [20], Germany [25], Switzerland [21], Wales [27], Canada [23], and the United States [28, 32]. A full overview is presented in Table 2.

**Table 2:** Time to treatment and changes in treatment modalities in the COVID-19 group compared the non-COVID-19 group.

Study	Site	Interval	Relation	Quantity (non-COVID-19 vs COVID-19)	*P*-value	Change in treatment modality
***Eastern European studies***						
Gršić et al.	Oral Oral Larynx Larynx	Symptom to specialist Specialist to treatment Specialist to treatment Symptom to specialist	PR PR PR NR	37.5 days vs 60 days (difference: +22.5 days) 26 days vs 37 days (difference: +11 days) 21.5 vs 31.5 days (difference: +10 days) 60 days vs 90 days (difference: +30 days)	0.019* 0.006* 0.001* 0.122	Not reported
Tevetoğlu et al.	Oral + Larynx	Symptom to specialist Specialist to treatment	PR NR	16.6 days vs 19.01 days (difference: +2.41 days) 2.5 days vs 2.9 days (difference: +0.4 days)	0.049* 0.06	Not reported
***Western European studies***						
Zubair et al.	HNC	Referral to specialist Referral to treatment Diagnosis to treatment	NR PR NR	7.1 days vs 11.9 days (difference: +4.8 days) 49.23 vs 72.5 days (difference: +23.27 days) 24.7 days vs 29.2 days (difference: +4.5 days)	0.068 0.027* 0.58	Not reported
Heckel et al.	HNC	Diagnosis to treatment	IR	23 days vs 19.5 (difference: –3.5 days)	0.013*	No change in treatment modality
Metzger et al.	Oral	Specialist to treatment	PR	35 days vs 45 days (difference: +10 days)	0.04*	No change in treatment modality
Kourtidis et al.	HNSCC	Symptom to diagnosis Diagnosis to treatment	NR NR	9.5 days vs 15 days (difference: +5.5 days) 3 days vs 3.2 days (difference: +0.2 days)	0.054 0.264	Not reported
Heimes et al.	Oral	Time to intervention	NR	22.99 days vs 26.66 days (difference: +3.67 days)	*p* > 0.05	Not reported
Schoonbeek et al.	HNC	Specialist to treatment Biopsy to treatment	IR IR	31 days vs 26 days (difference: –5 days) 37 days vs 30 days (difference: –7 days)	*p* < 0.001* *p* < 0.001*	No change in treatment modality
Drake et al.	HNC	Referral to diagnosis Referral to treatment	NR NR	No overall data No overall data		
Meerwein et al.	Sinonasal + nasopharynx	Symptom to biopsy Symptom to treatment Referral to treatment	NR NR IR	123 vs 129 days (difference: +6 days) 137 days vs 139 days (difference: +2 days) 18 days vs 11 days (difference: –7 days)	0.17 0.60 0.02*	Not reported
Abelardo et al.	HNC	Referral to specialist Referral to MDT Referral to treatment	IR NR NR	9.5 days vs 8 days (difference: –1.5 days) 41.5 days vs 35.5 days (difference: –6 days 78 days vs 68 days (difference: –10 days)	< 0.01* 0.40 0.16	Not reported
***Southern European studies***						
Lucidi et al.	HNC	Specialist to treatment	NR	47.6 days vs 44 days (difference: –3.6 days)	*p* > 0.05	No change in treatment modality
***Northern American studies***						
Psycharis et al.	HNC	Specialist to MDT Specialist to treatment Biopsy to diagnosis	IR IR NR	38 days vs 25.1 days (difference: –12.9 days) 76.6 days vs 48.7 days (difference: –27.9 days) 14.1 days vs 9.9 days (difference: –4.2 days)	0.0001* 0.001* 0.142	No change in treatment modality
Solis et al.	HNSCC	Symptom to specialist Biopsy to treatment Specialist to treatment Scan to treatment Diagnosis (elsewhere) to first visit	NR NR NR NR NR	133 days vs 112 days (difference: –21 days) 53 days vs 52 days (difference: –1 day) 29 days vs 27 days (difference: –2 days) 42 days vs 40 days (difference: –2 days) 25 days vs 27 days (difference: 2 days)	0.483 0.737 0.310 0.126 0.938	Not reported
Yao et al.	HNC	Suspicion to diagnosis Suspicion to staging Diagnosis to treatment	PR NR NR	COVID-19 group less likely to be diagnosed (HR = 0.54) COVID-19 group more likely to be diagnosed (HR = 1.01) COVID-19 group more likely to be diagnosed (HR = 1.55)	0.02* > 0.9 0.12	
Kiong et al.	HNC	Symptom to specialist Diagnosis (elsewhere) to first visit First visit to MDT	NR NR NR	12 weeks vs 12 weeks (difference: 0 weeks) 20 days vs 25 days (difference: +5 days) 2 days vs 2 days (difference: 0 days)	0.391 0.133 0.507	Not reported
Tasoulas et al.	HNC	Diagnosis to treatment	IR	46 days vs 43 days (difference: –3 days)	(95% CI: 46–47) vs (95% CI: 42–43)*	
Stevens et al.	HNSCC	Referral to specialist Symptom to specialist	IR NR	11 days vs 8 days (difference: –3 days) 6.82 weeks vs 6.54 weeks (difference: –0.28 weeks)	0.003* 0.872	No change in treatment modality
***Asian studies***						
Yang et al.	Nasopharynx	Pathological consultation report Report from biopsies Imaging examination Radiotherapy immobilization. and simulation Validation of position and plan Initiation of treatment	NR PR PR PR PR PR	3 days vs 2 days (difference: –1 day) 5 days vs 15 days (difference: +10 days) 1 day vs 8 days (difference: +7 days) 3.5 days vs 16.5 days (difference: +13 days) 20 days vs 61 days (difference: +41 days) 28 days vs 36 days (difference: +8 days)	0.111 0.012* > 0.001* > 0.001* > 0.001* 0.005*	Not reported

This table shows the difference in time to treatment when comparing the non-COVID-19 group with the COVID-19 group across each examined interval.

HNC: all head and neck cancers; HNSCC: head and neck squamous cell carcinomas; NR: no relation (neither significant increase nor decrease in time to treatment during the COVID-19 period); HR: hazard ratio; PR: positive relation (significant increase in time to treatment during COVID-19 period); IR: inverse relation (significant decrease in time to treatment during the COVID-19 period); MDT: multidisciplinary team conference; COVID-19: coronavirus disease 2019.

* Significant value.

### Changes in treatment regimens

Six studies reported on treatment regimens, and none found chances in treatment regimens used in the COVID-19 groups [19, 20, 23, 25, 26, 32].

### Stage, TNM classification, and tumor size

Fifteen studies [17–26, 31–35] reported on oncologic outcomes (*n* = 13,625), and none found a decrease in oncologic burden during the COVID-19 period. Eight studies found no significant difference in oncologic outcomes (*n* = 11,890) [17, 18, 20, 21, 23, 25, 31, 34], and seven studies observed an increase in at least one of the oncologic parameters (*n* = 1735) [19, 22, 24, 26, 32–35].

Ten studies reported on UICC stage [17–26, 31, 33, 34], and nine found no significant differences [17, 18, 20, 21, 25, 26, 31–34]. Lucidi et al. found that average UICC stage was higher in the COVID-19 group compared to the non-COVID-19 group (*n* = 265). They did not further assess T-, N-, and M-stage [19].

T-classification was assessed in 11 studies [17–26, 32–35], and seven found no significant relation [17, 21, 23, 25, 32, 34, 35]. Four studies found an increased prevalence of T3/T4 tumors in the COVID-19 group [22, 24, 26, 33]. Tevetoğlu et al. observed an increase from 28 to 53% in the COVID-19 period (*p* = 0.02, *n* = 116) [22]. Similar increases were found by Metzger et al. (36–52%, *p* = 0.046, *n* = 624) [26], Solis et al. (40.3–61.7%, *p* = 0.02, *n* = 137) [24], and Kiong et al. (39.4–52%, *p* = 0.03, *n* = 231) [33]. Two of the studies further investigated primary tumor size; Solis et al. found an increased median tumor size from 3.0 cm in the non-COVID-19 group compared to 4.5 cm in the COVID-19 group [24]. Similarly, Kiong et al. found an increased mean tumor size from 2.5 cm in the non-COVID-19 group to 2.9 cm in the COVID-19 group [33]. N-classification was assessed in the same 11 studies as T-classification [17, 21–26, 32–35], and 10 found no significant relation [17, 21–26, 33–35]. Stevens et al. identified an increased risk for patients presenting with nodal metastases in the COVID-19 group (adjusted odds ratio 1.8, *p* = 0.03) (*n* = 268) [32]. The presence of patients with metastatic disease at time of diagnosis was assessed in eight studies [17, 21, 23–25, 32, 33, 35], and seven found no relation [17, 21, 23–25, 32, 33]. Kourtidis et al. observed an increased frequency of metastatic disease (0% vs 10%, *p* = 0.022) in the COVID-19 group compared to the non-COVID-19 group (*n* = 94) [35]. Among the six studies that found increases in T, N, or M classification [22, 24, 26, 32, 33, 35], two further investigated the impact on UICC stage, and both found no significant effect [26, 33]. In total, increases in at least one oncologic parameter were observed in Germany [26, 35], Italy [19], Turkey [22], and the United States [24, 32, 33]. A full overview is presented in Table 3.

**Table 3:** Oncologic outcomes in the COVID-19 group compared to the non-COVID-19 group.

Study	Site	Oncologic outcome	Relation	Quantity (non-COVID-19 vs COVID-19)	*P*-value
***Eastern European studies***					
Gršić et al.	Oral + Larynx	Clinical TNM classification UICC numerical stage	NR NR		
Tevetoğlu et al.	Oral + Larynx	T classification N classification	PR NR	Proportion of T3/T4 tumors: 28% vs 53%	0.049*
***Western European studies***					
Zubair et al.	HNC	UICC numerical stage	NR		
Heckel et al.	HNC	Clinical TNM classification Pathologic TNM classification UICC numerical stage	NR NR NR		
Metzger et al.	Oral	Pathologic T-classification Pathologic N-classification UICC numerical stage	PR NR NR	Proportion of T3/T4 tumors: 36% vs 52%	0.046*
Kourtidis et al.	HNSCC	T classification N classification M classification	NR NR PR	0 (0%) vs 5 (10%)	0.022*
Heimes et al.	Oral	T and N classification UICC numerical stage	NR NR		
Schoonbeek et al.	HNC	UICC numerical stage	NR		
Drake et al.	HNC	No data with statistical testing			
Meerwein et al.	Sinonasal + nasopharynx	Clinical TNM classification UICC numerical stage	NR NR		
Abelardo et al.	HNC	No data			
***Southern European studies***					
Lucidi et al.	HNC	UICC numerical stage	PR	Average UICC stage higher in COVID-19 period	0.023*
***Northern American studies***					
Psycharis et al.	HNC	TNM classification	NR		
Solis et al.	HNSCC	T classification N classification M classification Median tumor size	PR NR NR PR	Proportion of T3/T4 tumors: 40.3% vs 61.7% 3.0 cm vs 4.5 cm	0.0244* 0.0002*
Yao et al.	HNC	No data			
Kiong et al.	HNC HNC HNC HNSCC only HNSCC only HNSCC only HNSCC only	TNM classification UICC numerical stage Mean size of tumor T classification N classification UICC numerical stage Mean tumor size	NR NR PR PR NR NR NR	2.5 cm vs 2.9 cm Proportion of T3/T4 tumors: vs 39.4% vs 52.0%	0.042* 0.025*
Tasoulas et al.	HNC	No data with statistical testing			
Stevens et al.	HNSCC	Clinical T classification Clinical N classification Clinical M classification Pathologic TNM classification Upstaging (C < P)	NR PR NR NR NR	Patients in COVID-period more likely to present with nodal metastases compared to non-COVID-19 (adjusted OR: 1.846)	0.028*
***Asian studies***					
Yang et al.	Nasopharynx	UICC numerical stage	NR		

This table shows the differences in oncologic outcomes (tumor stage, TNM classification, size, etc.), when comparing the non-COVID-19 group with the COVID-19 group. Quantity and *p*-values are indicated when there is a significant difference.

HNC: all head and neck cancers; HNSCC: head and neck squamous cell carcinomas; NR: no relation (neither significant increase nor decrease in oncologic outcome during the COVID-19 period); PR: positive relation (significant increase in oncologic outcome during the COVID-19 period); MDT: multidisciplinary team conference; TNM: Tumor Node and Metastasis; UICC: Union for International Cancer Control; COVID-19: coronavirus disease 2019.

* Significant value.

## Discussion

This systematic review investigating the impact of the COVID-19 pandemic on time to treatment intervals, treatment regimens, and tumor stage or size for HNC patients found modest variations in time to treatment, no effect on treatment regimens used, and a tendency toward presentation at a higher T-classification [22, 24, 26, 33]. To our knowledge, this is the first systematic review assessing the impact of COVID-19 on time to treatment.

The effect of COVID-19 on the time to treatment in HNC was divergent. Six studies reported an increase in waiting time within at least one interval [17, 18, 22, 26, 30, 31], while seven studies reported a decrease [20, 21, 23, 25, 27, 28, 32]. Across the examined intervals, on specific trends were noted. No relationship was observed between increased time to treatment and an increase in tumor stage, TNM classification, or size.

Most of the included patients found a decrease in time to treatment, which accounts for 20,779 out of 24,898 (83.5%), primarily due to the inclusion of the two largest studies [20, 28]. Overall, the pandemic resulted in marginal changes in time to treatment; among the studies that found increased time to treatment, the median increase was only 11 days across all intervals, suggesting that otorhinolaryngology and head & neck departments prioritized HNC care during the pandemic.

The heterogeneity of the results may be due in part to the differing impacts of the COVID-19 pandemic on various countries as well as disparities in healthcare organization and accessibility across nations [36, 37]. Furthermore, studies from Germany [25, 26] and the United States [28, 30, 32] showed opposing results, suggesting not only intercountry but also interregional differences.

Different factors could be associated with the increases in time to treatment observed [38]. First, the risk of viral exposure associated with visiting a medical facility may affect the time from onset of symptoms to seeking medical attention [39], and fear of overloading an already overwhelmed medical sector might contribute [38]. In this study, we found a tendency to increased T-classification [22, 24, 26, 33], which could indicate a delay in the pre-hospital phase, with patients presenting with symptoms later than optimal. Second, reallocation of resources might limit access to specialist consultations and diagnostic biopsies, thus increasing the time to diagnosis [38]. Third, anticipation of or actual shortage of critical care might lead to a reduction in surgical capacity, increasing the time to initiation of surgery [38]. While there have been indications of radiotherapy compensating for decreased surgical activity within other cancers [40], we did not observe any changes in the treatment modalities used during the COVID-19 pandemic [19, 20, 23, 25, 26, 32].

On the other hand, the COVID-19 pandemic could also be associated with the decreases in time to treatment observed [8–10, 41, 42]. Some hospitals experienced reductions in routine and elective procedures [8–10], and care could be diverted to treatments, which could not be postponed such as cancer treatment. Additionally, patients’ initial reluctance to seek medical attention might result in subsequent presentation at a more advanced T-stage, as indicated in the studies [22, 24, 26, 33], thus requiring more urgent and rapid treatment. Since only five studies assessed pre-hospital time to treatment intervals [17, 22, 24, 32, 33], we were not able to draw further conclusions on the potential impact of pre-hospital delay. The two largest studies [20, 28] encompassing a total of 19,348 patients collectively (77.4% of all patients included) were both registry-based and showed a small reduction in time to treatment. However, neither of these included time intervals starting from the onset of symptoms.

Moreover, during the initial phases of the pandemic, incidence rates of numerous cancers, including HNC, declined in several countries – possibly due to the above-mentioned factors influencing patients’ healthcare-seeking behavior, reducing cancer patient volume [41–43].

While results on time to treatment were inconsistent, a tendency was observed with respect to oncologic outcomes. Seven of 15 studies observed an increase in at least one oncologic parameter during the COVID-19 pandemic [19, 22, 24, 26, 32, 33, 35]. However, only one study [19] observed an increase in overall stage, while nine studies did not find an effect on overall stage [17, 18, 20, 21, 25, 26, 31, 33, 34]. Four of 11 studies observed increased T-classification [22, 24, 26, 33]. Two studies assessed primary tumor size, and both found an increase during the COVID-19 pandemic [24, 33]. Only two studies observed an increase in an oncologic parameter and a simultaneous increase in time to treatment [22, 26], indicating that other factors may have played a role, e.g. delay in the pre-hospital phase.

Considering the close relationship between T-classification and disease prognosis [44], these results suggest worsened prognosis among patients diagnosed with HNC during the pandemic in some regions. However, we were not able to include survival outcomes in this study due to the recency of the pandemic. Nonetheless, a higher T-classification has other implications such as more extensive surgery, wider radiation fields, and increasing patient morbidity [45, 46].

This study is limited by the studies selected for analysis containing considerable variation in definitions of time to treatment intervals. This highlights the importance of more standardized definitions of time to treatment to increase comparability and generalizability. Also, the definition of COVID-19 periods as well as the subtypes of HNCs analyzed varied. Due to the recency of the pandemic, studies lack important clinical endpoints like 5-year survival rates, and comparison of COVID-19 and non-COVID-19 periods that are not analogous might be subject to seasonal variance of patient flow. Individual studies suffering from limitations including the inherent retrospective design with studies assessing time intervals beginning from the onset of symptoms might be subjected to recall bias. Also, the study by Tasoulas et al. [28] used the National Cancer Database, which might have incorporated patients from the four other American studies [24, 30, 32, 33]. Finally, variations in healthcare structures, the prevalence and severity of COVID-19 as well as discrepancies in restrictions imposed by distinct government authorities may influence medical systems differently. This complexity hinders broad conclusions applicable across diverse geographical areas.

In conclusion, this systematic review found that the impact of the COIVD-19 pandemic on time to treatment was heterogenous and subject to considerable intercountry and interregional variations. No change in treatment modalities used was observed. Consensus on definitions on time to treatment is required to enhance the overall generalizability. No significant impact on overall stage was observed, but a tendency toward a higher T-classification was observed in both Europe and the United States. In conclusion, otorhinolaryngology and head & neck departments seemed to have prioritized HNC care during the pandemic.

## Supplementary Material

The impact of the COVID-19 pandemic on time to treatment in head and neck cancer management: a systematic review

## Data Availability

Data are available on the PubMed and Embase databases.
