# Supplementary material for: The impact of the COVID-19 pandemic on time to treatment in head and neck cancer management: a systematic review
Source: Acta Oncol. 2025 Jan 28;64:41366. doi: 10.2340/1651-226X.2025.41366 (PMC11808816; doi:10.2340/1651-226X.2025.41366)
Supplement: The impact of the COVID-19 pandemic on time to treatment in head and neck cancer management: a systematic review [file AO-64-41366-s1.pdf]

Supplementary material has been published as submitted. It has not been copyedited, or typeset by Acta Oncologica

Search string, Embase.

#Search	Query	# Results Embase
#1	**"Time to treatment*" OR "Waiting time" OR "Time to treatment initiation" OR "TTI" OR "Treatment delay*" OR "treatment delays" OR "Delay in treatment*" OR "delays in treatment" OR "Delayed treatment*" OR "time to surgery" OR "door to treatment"**	117.877
#2	**Exp time to treatment /**	26.656
#3	#2 OR #3	117.877
#4	**Exp nose cancer /**<br>**Exp mouth cancer /**<br>**Exp larynx cancer /**<br>**Exp pharynx cancer /**<br>**Exp head and neck squamos cell carcinoma**	159.182
#5	**(Head OR Neck OR "upper aerodigestive tract" OR "uadt" OR Nose OR Nasal OR Sinonasal OR Paranasal OR sinuses OR Throat OR Pharynx OR Larynx OR laryngeal OR nasopharynx OR oral**	696.493

	**OR mouth OR oropharynx OR hypopharynx) AND (neoplasm OR cancer OR Tumor OR Tumour OR Carcinoma) OR HNSCC**	
#6	#4 OR #5	703182
#7	#6 AND #3. Limit to full text and Embase	1034
#8	#7. Publication year 2020-2023	237

Search string, PubMed

#Search	Query	# Results PubMed
#1	**"Time to treatment*" OR "Waiting time" OR "Time to treatment initiation" OR "TTI" OR "Treatment delay*" OR "treatment delays" OR "Delay in treatment*" OR "delays in treatment" OR "Delayed treatment*" OR "time to surgery" OR "door to treatment"**	36.068
#2	**"Time-to-Treatment"[Mesh]**	9.880
#3	#2 OR #3	36.068
#4	**"Squamous Cell Carcinoma of Head and Neck"[Mesh] OR "Pharyngeal Neoplasms"[Mesh] OR "Mouth Neoplasms"[Mesh] OR"Laryngeal Neoplasms"[Mesh] OR "Nose Neoplasms"[Mesh]**	158.459
#5	**(Head OR Neck OR "upper aerodigestive tract" OR "uadt" OR Nose OR Nasal OR Sinonasal OR Paranasal OR sinuses OR Throat OR Pharynx OR Larynx OR laryngeal OR nasopharynx OR oral OR mouth OR oropharynx OR hypopharynx) AND (neoplasm OR cancer OR Tumor OR**	502.340

	**Tumour OR Carcinoma) OR HNSCC**	
#6	#4 OR #5	526.547
#7	#6 AND #3	1.111
#8	#7. Publication year 2020-2023	382

Quality assessment, AXIS-tool

**Study** Gršić et al.	**Question**	**Yes**	**No**	**Don't know/ Comment**
Introduction				
1	Were the aims/objectives of the study clear?	X		
Methods				
2	Was the study design appropriate for the stated aim(s)?	X		
3	Was the sample size justified?	X		
4	Was the target/reference population clearly defined? (Is it clear who the research was about?)	X		
5	Was the sample frame taken from an appropriate population base so that it closely represented the target/reference population under investigation?		X	Reference population is HNC patients, samples are only from larynx and oral cancer
6	Was the selection process likely to select subjects/participants that were representative of the target/reference population under investigation?	X		
7	Were measures undertaken to address and categorise non-responders?		X	
8	Were the risk factor and outcome variables measured appropriate to the aims of the study?		X	No justification for COVID-19 period.
9	Were the risk factor and outcome variables measured correctly using instruments/measurements that had been trialled, piloted or published previously?		X	Outcome not trialled, piloted nor published
10	Is it clear what was used to determined statistical significance and/or precision estimates? (e.g. p-values, confidence intervals)	X		
11	Were the methods (including statistical methods) sufficiently described to enable them to be repeated?	X		
Results				
12	Were the basic data adequately described?		X	No adequate table 1
13	Does the response rate raise concerns about non-response bias?	X		
14	If appropriate, was information about non-responders described?			
15	Were the results internally consistent?		X	Table 4 inadequate.
16	Were the results presented for all the analyses described in the methods?	X		
Discussion				
17	Were the authors' discussions and conclusions justified by the results?	X		
18	Were the limitations of the study discussed?	X		
Other				
19	Were there any funding sources or conflicts of interest that may affect the authors' interpretation of the results?		X	Section not included.
20	Was ethical approval or consent of participants attained?		X	Section not included.

**Study** Zubair et al.	**Question**	**Yes**	**No**	**Don't know/ Comment**
Introduction				
1	Were the aims/objectives of the study clear?	X		
Methods				
2	Was the study design appropriate for the stated aim(s)?	X		
3	Was the sample size justified?	X		
4	Was the target/reference population clearly defined? (Is it clear who the research was about?)	X		
5	Was the sample frame taken from an appropriate population base so that it closely represented the target/reference population under investigation?	X		
6	Was the selection process likely to select subjects/participants that were representative of the target/reference population under investigation?	X		
7	Were measures undertaken to address and categorise non-responders?		X	
8	Were the risk factor and outcome variables measured appropriate to the aims of the study?		X	Inadequate justification for decision of COVID-19 period.
9	Were the risk factor and outcome variables measured correctly using instruments/measurements that had been trialled, piloted or published previously?		X	Outcome not trialled, piloted nor published
10	Is it clear what was used to determined statistical significance and/or precision estimates? (e.g. p-values, confidence intervals)	X		
11	Were the methods (including statistical methods) sufficiently described to enable them to be repeated?	X		
Results				
12	Were the basic data adequately described?		X	Inadequate table 1
13	Does the response rate raise concerns about non-response bias?	X		
14	If appropriate, was information about non-responders described?			
15	Were the results internally consistent?	X		
16	Were the results presented for all the analyses described in the methods?	X		
Discussion				
17	Were the authors' discussions and conclusions justified by the results?	X		
18	Were the limitations of the study discussed?		X	
Other				
19	Were there any funding sources or conflicts of interest that may affect the authors' interpretation of the results?			Section not included
20	Was ethical approval or consent of participants attained?		X	Section not included

**Study** Lucidi et al.	**Question**	**Yes**	**No**	**Don't know/ Comment**
Introduction				
1	Were the aims/objectives of the study clear?	X		
Methods				
2	Was the study design appropriate for the stated aim(s)?	X		
3	Was the sample size justified?	X		
4	Was the target/reference population clearly defined? (Is it clear who the research was about?)	X		
5	Was the sample frame taken from an appropriate population base so that it closely represented the target/reference population under investigation?	X		
6	Was the selection process likely to select subjects/participants that were representative of the target/reference population under investigation?	X		
7	Were measures undertaken to address and categorise non-responders?	X		
8	Were the risk factor and outcome variables measured appropriate to the aims of the study?	X		Justification of COVID-19 period. Time intervals clearly defined.
9	Were the risk factor and outcome variables measured correctly using instruments/measurements that had been trialled, piloted or published previously?		X	Outcome not trialled, piloted nor published
10	Is it clear what was used to determined statistical significance and/or precision estimates? (e.g. p-values, confidence intervals)	X		
11	Were the methods (including statistical methods) sufficiently described to enable them to be repeated?	X		
Results				
12	Were the basic data adequately described?		X	Inadequate table 1
13	Does the response rate raise concerns about non-response bias?	X		
14	If appropriate, was information about non-responders described?			
15	Were the results internally consistent?	X		
16	Were the results presented for all the analyses described in the methods?		X	Inadequate data for UICC tumor stage
Discussion				
17	Were the authors' discussions and conclusions justified by the results?	X		
18	Were the limitations of the study discussed?		X	
Other				
19	Were there any funding sources or conflicts of interest that may affect the authors' interpretation of the results?		X	No conflicts of interest
20	Was ethical approval or consent of participants attained?		X	Section not included

**Study** Schoonbeek et. al	**Question**	**Yes**	**No**	**Don’t know/ Comment**
Introduction				
1	Were the aims/objectives of the study clear?	X		
Methods				
2	Was the study design appropriate for the stated aim(s)?	X		
3	Was the sample size justified?	X		
4	Was the target/reference population clearly defined? (Is it clear who the research was about?)	X		
5	Was the sample frame taken from an appropriate population base so that it closely represented the target/reference population under investigation?	X		
6	Was the selection process likely to select subjects/participants that were representative of the target/reference population under investigation?	X		
7	Were measures undertaken to address and categorise non-responders?	X		
8	Were the risk factor and outcome variables measured appropriate to the aims of the study?	X		
9	Were the risk factor and outcome variables measured correctly using instruments/measurements that had been trialled, piloted or published previously?	X		
10	Is it clear what was used to determined statistical significance and/or precision estimates? (e.g. p-values, confidence intervals)	X		
11	Were the methods (including statistical methods) sufficiently described to enable them to be repeated?	X		
Results				
12	Were the basic data adequately described?	X		
13	Does the response rate raise concerns about non-response bias?	X		
14	If appropriate, was information about non-responders described?			
15	Were the results internally consistent?	X		
16	Were the results presented for all the analyses described in the methods?	X		
Discussion				
17	Were the authors' discussions and conclusions justified by the results?	X		
18	Were the limitations of the study discussed?	X		
Other				
19	Were there any funding sources or conflicts of interest that may affect the authors’ interpretation of the results?		X	No conflicts of interest
20	Was ethical approval or consent of participants attained?	X		

Study Meerwein et a.	Question	Yes	No	Don't know/ Comment
Introduction				
1	Were the aims/objectives of the study clear?	X		
Methods				
2	Was the study design appropriate for the stated aim(s)?	X		
3	Was the sample size justified?	X		
4	Was the target/reference population clearly defined? (Is it clear who the research was about?)	X		
5	Was the sample frame taken from an appropriate population base so that it closely represented the target/reference population under investigation?	X		
6	Was the selection process likely to select subjects/participants that were representative of the target/reference population under investigation?	X		
7	Were measures undertaken to address and categorise non-responders?	X		
8	Were the risk factor and outcome variables measured appropriate to the aims of the study?		X	Inadequate justification for decision of COVID-19 period.
9	Were the risk factor and outcome variables measured correctly using instruments/measurements that had been trialled, piloted or published previously?		X	Outcome not trialled, piloted nor published
10	Is it clear what was used to determined statistical significance and/or precision estimates? (e.g. p-values, confidence intervals)	X		
11	Were the methods (including statistical methods) sufficiently described to enable them to be repeated?	X		
Results				
12	Were the basic data adequately described?	X		
13	Does the response rate raise concerns about non-response bias?	X		
14	If appropriate, was information about non-responders described?			
15	Were the results internally consistent?	X		
16	Were the results presented for all the analyses described in the methods?	X		
Discussion				
17	Were the authors' discussions and conclusions justified by the results?	X		
18	Were the limitations of the study discussed?	X		
Other				
19	Were there any funding sources or conflicts of interest that may affect the authors' interpretation of the results?		X	No conflicts of interest
20	Was ethical approval or consent of participants attained?	X		

**Study** Tevetoğlu et. al	**Question**	**Yes**	**No**	**Don’t know/ Comment**
Introduction				
1	Were the aims/objectives of the study clear?	X		
Methods				
2	Was the study design appropriate for the stated aim(s)?	X		
3	Was the sample size justified?	X		
4	Was the target/reference population clearly defined? (Is it clear who the research was about?)	X		
5	Was the sample frame taken from an appropriate population base so that it closely represented the target/reference population under investigation?		X	HNC vs Laryx, Oral
6	Was the selection process likely to select subjects/participants that were representative of the target/reference population under investigation?		X	
7	Were measures undertaken to address and categorise non-responders?		X	
8	Were the risk factor and outcome variables measured appropriate to the aims of the study?	X		
9	Were the risk factor and outcome variables measured correctly using instruments/measurements that had been trialled, piloted or published previously?		X	Outcome not trialled, piloted nor published
10	Is it clear what was used to determined statistical significance and/or precision estimates? (e.g. p-values, confidence intervals)	X		
11	Were the methods (including statistical methods) sufficiently described to enable them to be repeated?	X		
Results				
12	Were the basic data adequately described?		X	Inadequate table 1
13	Does the response rate raise concerns about non-response bias?	X		
14	If appropriate, was information about non-responders described?			
15	Were the results internally consistent?	X		
16	Were the results presented for all the analyses described in the methods?	X		
Discussion				
17	Were the authors' discussions and conclusions justified by the results?	X		
18	Were the limitations of the study discussed?			
Other				
19	Were there any funding sources or conflicts of interest that may affect the authors’ interpretation of the results?		X	
20	Was ethical approval or consent of participants attained?	X		

**Study** Psycharis et. al	**Question**	**Yes**	**No**	**Don't know/ Comment**
Introduction				
1	Were the aims/objectives of the study clear?	X		
Methods				
2	Was the study design appropriate for the stated aim(s)?	X		
3	Was the sample size justified?	X		
4	Was the target/reference population clearly defined? (Is it clear who the research was about?)	X		
5	Was the sample frame taken from an appropriate population base so that it closely represented the target/reference population under investigation?	X		
6	Was the selection process likely to select subjects/participants that were representative of the target/reference population under investigation?	X		
7	Were measures undertaken to address and categorise non-responders?		X	
8	Were the risk factor and outcome variables measured appropriate to the aims of the study?	X		
9	Were the risk factor and outcome variables measured correctly using instruments/measurements that had been trialled, piloted or published previously?		X	Outcome not trialled, piloted nor published
10	Is it clear what was used to determined statistical significance and/or precision estimates? (e.g. p-values, confidence intervals)	X		
11	Were the methods (including statistical methods) sufficiently described to enable them to be repeated?	X		
Results				
12	Were the basic data adequately described?	X		
13	Does the response rate raise concerns about non-response bias?	X		
14	If appropriate, was information about non-responders described?			
15	Were the results internally consistent?	X		
16	Were the results presented for all the analyses described in the methods?	X		
Discussion				
17	Were the authors' discussions and conclusions justified by the results?	X		
18	Were the limitations of the study discussed?	X		
Other				
19	Were there any funding sources or conflicts of interest that may affect the authors' interpretation of the results?		X	
20	Was ethical approval or consent of participants attained?	X		

Study Solis et al.	Question	Yes	No	Don't know/ Comment
Introduction				
1	Were the aims/objectives of the study clear?	X		
Methods				
2	Was the study design appropriate for the stated aim(s)?	X		
3	Was the sample size justified?	X		
4	Was the target/reference population clearly defined? (Is it clear who the research was about?)	X		
5	Was the sample frame taken from an appropriate population base so that it closely represented the target/reference population under investigation?	X		
6	Was the selection process likely to select subjects/participants that were representative of the target/reference population under investigation?	X		
7	Were measures undertaken to address and categorise non-responders?	X		
8	Were the risk factor and outcome variables measured appropriate to the aims of the study?	X		
9	Were the risk factor and outcome variables measured correctly using instruments/measurements that had been trialled, piloted or published previously?		X	Outcome not trialled, piloted nor published
10	Is it clear what was used to determined statistical significance and/or precision estimates? (e.g. p-values, confidence intervals)	X		
11	Were the methods (including statistical methods) sufficiently described to enable them to be repeated?	X		
Results				
12	Were the basic data adequately described?	X		
13	Does the response rate raise concerns about non-response bias?	X		
14	If appropriate, was information about non-responders described?			
15	Were the results internally consistent?	X		
16	Were the results presented for all the analyses described in the methods?	X		
Discussion				
17	Were the authors' discussions and conclusions justified by the results?	X		
18	Were the limitations of the study discussed?	X		
Other				
19	Were there any funding sources or conflicts of interest that may affect the authors' interpretation of the results?		X	
20	Was ethical approval or consent of participants attained?	X		

Study Heckel et al.	Question	Yes	No	Don't know/ Comment
Introduction				
1	Were the aims/objectives of the study clear?	X		
Methods				
2	Was the study design appropriate for the stated aim(s)?	X		
3	Was the sample size justified?	X		
4	Was the target/reference population clearly defined? (Is it clear who the research was about?)	X		
5	Was the sample frame taken from an appropriate population base so that it closely represented the target/reference population under investigation?	X		
6	Was the selection process likely to select subjects/participants that were representative of the target/reference population under investigation?	X		
7	Were measures undertaken to address and categorise non-responders?	X		
8	Were the risk factor and outcome variables measured appropriate to the aims of the study?		X	Inadequate justification for definition of COVID-19 period
9	Were the risk factor and outcome variables measured correctly using instruments/measurements that had been trialled, piloted or published previously?		X	Outcome not trialled, piloted nor published
10	Is it clear what was used to determined statistical significance and/or precision estimates? (e.g. p-values, confidence intervals)	X		
11	Were the methods (including statistical methods) sufficiently described to enable them to be repeated?	X		
Results				
12	Were the basic data adequately described?	X		
13	Does the response rate raise concerns about non-response bias?		X	
14	If appropriate, was information about non-responders described?	X		
15	Were the results internally consistent?	X		
16	Were the results presented for all the analyses described in the methods?	X		
Discussion				
17	Were the authors' discussions and conclusions justified by the results?	X		
18	Were the limitations of the study discussed?	X		
Other				
19	Were there any funding sources or conflicts of interest that may affect the authors' interpretation of the results?		X	
20	Was ethical approval or consent of participants attained?	X		

Study Metzger et al.	Question	Yes	No	Don't know/ Comment
Introduction				
1	Were the aims/objectives of the study clear?	X		
Methods				
2	Was the study design appropriate for the stated aim(s)?	X		
3	Was the sample size justified?	X		
4	Was the target/reference population clearly defined? (Is it clear who the research was about?)	X		
5	Was the sample frame taken from an appropriate population base so that it closely represented the target/reference population under investigation?	X		
6	Was the selection process likely to select subjects/participants that were representative of the target/reference population under investigation?	X		
7	Were measures undertaken to address and categorise non-responders?		X	
8	Were the risk factor and outcome variables measured appropriate to the aims of the study?		X	Argues for using 2020 as a COVID-19 year
9	Were the risk factor and outcome variables measured correctly using instruments/measurements that had been trialled, piloted or published previously?		X	Outcome not trialled, piloted nor published
10	Is it clear what was used to determined statistical significance and/or precision estimates? (e.g. p-values, confidence intervals)	X		
11	Were the methods (including statistical methods) sufficiently described to enable them to be repeated?	X		
Results				
12	Were the basic data adequately described?	X		
13	Does the response rate raise concerns about non-response bias?	X		
14	If appropriate, was information about non-responders described?			
15	Were the results internally consistent?	X		
16	Were the results presented for all the analyses described in the methods?	X		
Discussion				
17	Were the authors' discussions and conclusions justified by the results?	X		
18	Were the limitations of the study discussed?	X		
Other				
19	Were there any funding sources or conflicts of interest that may affect the authors' interpretation of the results?		X	
20	Was ethical approval or consent of participants attained?	X		

Study Abelardo et al.	Question	Yes	No	Don’t know/ Comment
Introduction				
1	Were the aims/objectives of the study clear?	X		
Methods				
2	Was the study design appropriate for the stated aim(s)?	X		
3	Was the sample size justified?	X		
4	Was the target/reference population clearly defined? (Is it clear who the research was about?)	X		
5	Was the sample frame taken from an appropriate population base so that it closely represented the target/reference population under investigation?	X		
6	Was the selection process likely to select subjects/participants that were representative of the target/reference population under investigation?		X	Selection process not described with inclusion and exclusion criteria
7	Were measures undertaken to address and categorize non-responders?		X	
8	Were the risk factor and outcome variables measured appropriate to the aims of the study?	X		
9	Were the risk factor and outcome variables measured correctly using instruments/measurements that had been trialled, piloted or published previously?		X	Outcome not trialled, piloted nor published
10	Is it clear what was used to determined statistical significance and/or precision estimates? (e.g. p-values, confidence intervals)		X	Level of significance not mentioned
11	Were the methods (including statistical methods) sufficiently described to enable them to be repeated?	X		
Results				
12	Were the basic data adequately described?	X		
13	Does the response rate raise concerns about non-response bias?	X		
14	If appropriate, was information about non-responders described?			
15	Were the results internally consistent?	X		
16	Were the results presented for all the analyses described in the methods?	X		
Discussion				
17	Were the authors' discussions and conclusions justified by the results?	X		
18	Were the limitations of the study discussed?		X	
Other				
19	Were there any funding sources or conflicts of interest that may affect the authors’ interpretation of the results?			Section not included.
20	Was ethical approval or consent of participants attained?		X	Section not included.

Study Ref: Tasoulas et al.	Question	Yes	No	Don’t know/ Comment
Introduction				
1	Were the aims/objectives of the study clear?	X		
Methods				
2	Was the study design appropriate for the stated aim(s)?	X		
3	Was the sample size justified?	X		
4	Was the target/reference population clearly defined? (Is it clear who the research was about?)	X		
5	Was the sample frame taken from an appropriate population base so that it closely represented the target/reference population under investigation?	X		
6	Was the selection process likely to select subjects/participants that were representative of the target/reference population under investigation?	X		
7	Were measures undertaken to address and categorise non-responders?	X		
8	Were the risk factor and outcome variables measured appropriate to the aims of the study?		X	Inadequate justification for definition of COVID-19 period
9	Were the risk factor and outcome variables measured correctly using instruments/measurements that had been trialled, piloted or published previously?		X	Outcome not trialled, piloted nor published
10	Is it clear what was used to determined statistical significance and/or precision estimates? (e.g. p-values, confidence intervals)	X		
11	Were the methods (including statistical methods) sufficiently described to enable them to be repeated?	X		
Results				
12	Were the basic data adequately described?		X	2019 used as comparator to 2020 data. No basic data on 2019 alone.
13	Does the response rate raise concerns about non-response bias?	X		
14	If appropriate, was information about non-responders described?			
15	Were the results internally consistent?	X		
16	Were the results presented for all the analyses described in the methods?	X		
Discussion				
17	Were the authors' discussions and conclusions justified by the results?	X		
18	Were the limitations of the study discussed?	X		
Other				
19	Were there any funding sources or conflicts of interest that may affect the authors’ interpretation of the results?		X	
20	Was ethical approval or consent of participants attained?	X		

**Study** Drake et. al	**Question**	**Yes**	**No**	**Don't know/ Comment**
Introduction				
1	Were the aims/objectives of the study clear?	X		
Methods				
2	Was the study design appropriate for the stated aim(s)?	X		
3	Was the sample size justified?	X		
4	Was the target/reference population clearly defined? (Is it clear who the research was about?)	X		
5	Was the sample frame taken from an appropriate population base so that it closely represented the target/reference population under investigation?	X		
6	Was the selection process likely to select subjects/participants that were representative of the target/reference population under investigation?	X		
7	Were measures undertaken to address and categorise non-responders?	X		
8	Were the risk factor and outcome variables measured appropriate to the aims of the study?	X		
9	Were the risk factor and outcome variables measured correctly using instruments/measurements that had been trialled, piloted or published previously?		X	Outcome not trialled, piloted nor published
10	Is it clear what was used to determined statistical significance and/or precision estimates? (e.g. p-values, confidence intervals)		X	Statistical methods not described in methods section
11	Were the methods (including statistical methods) sufficiently described to enable them to be repeated?		X	Statistical significance not defined.
Results				
12	Were the basic data adequately described?	X		
13	Does the response rate raise concerns about non-response bias?	X		
14	If appropriate, was information about non-responders described?			
15	Were the results internally consistent?	X		
16	Were the results presented for all the analyses described in the methods?	X		
Discussion				
17	Were the authors' discussions and conclusions justified by the results?	X		
18	Were the limitations of the study discussed?		X	
Other				
19	Were there any funding sources or conflicts of interest that may affect the authors' interpretation of the results?		X	
20	Was ethical approval or consent of participants attained?	X		

Study Yao et al.	Question	Yes	No	Don't know/ Comment
Introduction				
1	Were the aims/objectives of the study clear?	X		
Methods				
2	Was the study design appropriate for the stated aim(s)?	X		
3	Was the sample size justified?	X		
4	Was the target/reference population clearly defined? (Is it clear who the research was about?)	X		
5	Was the sample frame taken from an appropriate population base so that it closely represented the target/reference population under investigation?		X	Name of tertiary center not mentioned
6	Was the selection process likely to select subjects/participants that were representative of the target/reference population under investigation?		X	Name of tertiary center not mentioned
7	Were measures undertaken to address and categorise non-responders?	X		
8	Were the risk factor and outcome variables measured appropriate to the aims of the study?	X		Suspicion to X. Author identifies as key clinical time point.
9	Were the risk factor and outcome variables measured correctly using instruments/measurements that had been trialled, piloted or published previously?		X	Outcome not trialled, piloted nor published
10	Is it clear what was used to determined statistical significance and/or precision estimates? (e.g. p-values, confidence intervals)	X		
11	Were the methods (including statistical methods) sufficiently described to enable them to be repeated?	X		
Results				
12	Were the basic data adequately described?	X		
13	Does the response rate raise concerns about non-response bias?	X		
14	If appropriate, was information about non-responders described?			
15	Were the results internally consistent?	X		
16	Were the results presented for all the analyses described in the methods?	X		
Discussion				
17	Were the authors' discussions and conclusions justified by the results?	X		
18	Were the limitations of the study discussed?		X	
Other				
19	Were there any funding sources or conflicts of interest that may affect the authors' interpretation of the results?		X	
20	Was ethical approval or consent of participants attained?	X		

**Study** Yang et al.	**Question**	**Yes**	**No**	**Don't know/ Comment**
Introduction				
1	Were the aims/objectives of the study clear?	X		
Methods				
2	Was the study design appropriate for the stated aim(s)?	X		
3	Was the sample size justified?	X		
4	Was the target/reference population clearly defined? (Is it clear who the research was about?)	X		
5	Was the sample frame taken from an appropriate population base so that it closely represented the target/reference population under investigation?	X		
6	Was the selection process likely to select subjects/participants that were representative of the target/reference population under investigation?	X		
7	Were measures undertaken to address and categorise non-responders?		X	
8	Were the risk factor and outcome variables measured appropriate to the aims of the study?	X		
9	Were the risk factor and outcome variables measured correctly using instruments/measurements that had been trialled, piloted or published previously?		X	Outcome not trialled, piloted nor published
10	Is it clear what was used to determined statistical significance and/or precision estimates? (e.g. p-values, confidence intervals)	X		
11	Were the methods (including statistical methods) sufficiently described to enable them to be repeated?	X		
Results				
12	Were the basic data adequately described?	X		
13	Does the response rate raise concerns about non-response bias?	X		
14	If appropriate, was information about non-responders described?			
15	Were the results internally consistent?	X		
16	Were the results presented for all the analyses described in the methods?	X		
Discussion				
17	Were the authors' discussions and conclusions justified by the results?	X		
18	Were the limitations of the study discussed?	X		
Other				
19	Were there any funding sources or conflicts of interest that may affect the authors' interpretation of the results?		X	
20	Was ethical approval or consent of participants attained?	X		

**Study** Stevens et al.	**Question**	**Yes**	**No**	**Don't know/ Comment**
Introduction				
1	Were the aims/objectives of the study clear?	X		
Methods				
2	Was the study design appropriate for the stated aim(s)?	X		
3	Was the sample size justified?	X		
4	Was the target/reference population clearly defined? (Is it clear who the research was about?)	X		
5	Was the sample frame taken from an appropriate population base so that it closely represented the target/reference population under investigation?	X		
6	Was the selection process likely to select subjects/participants that were representative of the target/reference population under investigation?	X		
7	Were measures undertaken to address and categorise non-responders?	X		
8	Were the risk factor and outcome variables measured appropriate to the aims of the study?	X		
9	Were the risk factor and outcome variables measured correctly using instruments/measurements that had been trialled, piloted or published previously?		X	Outcome not trialled, piloted nor published
10	Is it clear what was used to determined statistical significance and/or precision estimates? (e.g. p-values, confidence intervals)	X		
11	Were the methods (including statistical methods) sufficiently described to enable them to be repeated?	X		
Results				
12	Were the basic data adequately described?	X		
13	Does the response rate raise concerns about non-response bias?	X		Assessed in study. 49/268 patients excluded.
14	If appropriate, was information about non-responders described?	X		
15	Were the results internally consistent?	X		
16	Were the results presented for all the analyses described in the methods?	X		
Discussion				
17	Were the authors' discussions and conclusions justified by the results?	X		
18	Were the limitations of the study discussed?	X		
Other				
19	Were there any funding sources or conflicts of interest that may affect the authors' interpretation of the results?		X	
20	Was ethical approval or consent of participants attained?	X		

Study Kiong et al.	Question	Yes	No	Don't know/ Comment
Introduction				
1	Were the aims/objectives of the study clear?	X		
Methods				
2	Was the study design appropriate for the stated aim(s)?	X		
3	Was the sample size justified?	X		
4	Was the target/reference population clearly defined? (Is it clear who the research was about?)	X		
5	Was the sample frame taken from an appropriate population base so that it closely represented the target/reference population under investigation?	X		
6	Was the selection process likely to select subjects/participants that were representative of the target/reference population under investigation?	X		
7	Were measures undertaken to address and categorise non-responders?		X	
8	Were the risk factor and outcome variables measured appropriate to the aims of the study?	X		
9	Were the risk factor and outcome variables measured correctly using instruments/measurements that had been trialled, piloted or published previously?		X	Outcome not trialled, piloted nor published
10	Is it clear what was used to determined statistical significance and/or precision estimates? (e.g. p-values, confidence intervals)	X		
11	Were the methods (including statistical methods) sufficiently described to enable them to be repeated?	X		
Results				
12	Were the basic data adequately described?	X		
13	Does the response rate raise concerns about non-response bias?	X		
14	If appropriate, was information about non-responders described?			
15	Were the results internally consistent?	X		
16	Were the results presented for all the analyses described in the methods?	X		
Discussion				
17	Were the authors' discussions and conclusions justified by the results?	X		
18	Were the limitations of the study discussed?	X		
Other				
19	Were there any funding sources or conflicts of interest that may affect the authors' interpretation of the results?			Section not included
20	Was ethical approval or consent of participants attained?	X		

**Study** Heimes et al.	**Question**	**Yes**	**No**	**Don't know/ Comment**
Introduction				
1	Were the aims/objectives of the study clear?		X	
Methods				
2	Was the study design appropriate for the stated aim(s)?	X		
3	Was the sample size justified?	X		
4	Was the target/reference population clearly defined? (Is it clear who the research was about?)	X		
5	Was the sample frame taken from an appropriate population base so that it closely represented the target/reference population under investigation?	X		
6	Was the selection process likely to select subjects/participants that were representative of the target/reference population under investigation?	X		
7	Were measures undertaken to address and categorise non-responders?		X	
8	Were the risk factor and outcome variables measured appropriate to the aims of the study?		X	Time to treatment interval not defined.
9	Were the risk factor and outcome variables measured correctly using instruments/measurements that had been trialled, piloted or published previously?		X	Outcome not trialled, piloted nor published
10	Is it clear what was used to determined statistical significance and/or precision estimates? (e.g. p-values, confidence intervals)	X		
11	Were the methods (including statistical methods) sufficiently described to enable them to be repeated?	X		
Results				
12	Were the basic data adequately described?		X	Inadequate table 1
13	Does the response rate raise concerns about non-response bias?	X		
14	If appropriate, was information about non-responders described?			
15	Were the results internally consistent?	X		
16	Were the results presented for all the analyses described in the methods?		X	
Discussion				
17	Were the authors' discussions and conclusions justified by the results?	X		
18	Were the limitations of the study discussed?	X		
Other				
19	Were there any funding sources or conflicts of interest that may affect the authors' interpretation of the results?		X	
20	Was ethical approval or consent of participants attained?	X		

Study Kourtidis et al.	Question	Yes	No	Don't know/ Comment
Introduction				
1	Were the aims/objectives of the study clear?	X		
Methods				
2	Was the study design appropriate for the stated aim(s)?	X		
3	Was the sample size justified?	X		
4	Was the target/reference population clearly defined? (Is it clear who the research was about?)	X		
5	Was the sample frame taken from an appropriate population base so that it closely represented the target/reference population under investigation?	X		
6	Was the selection process likely to select subjects/participants that were representative of the target/reference population under investigation?	X		
7	Were measures undertaken to address and categorise non-responders?		X	
8	Were the risk factor and outcome variables measured appropriate to the aims of the study?	X		
9	Were the risk factor and outcome variables measured correctly using instruments/measurements that had been trialled, piloted or published previously?			Outcome not trialled, piloted nor published
10	Is it clear what was used to determined statistical significance and/or precision estimates? (e.g. p-values, confidence intervals)	X		
11	Were the methods (including statistical methods) sufficiently described to enable them to be repeated?	X		
Results				
12	Were the basic data adequately described?	X		
13	Does the response rate raise concerns about non-response bias?	X		
14	If appropriate, was information about non-responders described?			
15	Were the results internally consistent?	X		
16	Were the results presented for all the analyses described in the methods?	X		
Discussion				
17	Were the authors' discussions and conclusions justified by the results?	X		
18	Were the limitations of the study discussed?	X		
Other				
19	Were there any funding sources or conflicts of interest that may affect the authors' interpretation of the results?		X	
20	Was ethical approval or consent of participants attained?	X		
